# Mental health and use of health care services in opioid-exposed school-aged children compared to foster children

**DOI:** 10.1007/s00787-021-01728-3

**Published:** 2021-02-16

**Authors:** Monica Sarfi, Marie Eikemo, Gabrielle K. Welle-Strand, Ashley Elizabeth Muller, Stine Lehmann

**Affiliations:** 1 SERAF-Norwegian Centre for Addiction Research, University of Oslo, Blindern, Box 1039, 0315 Oslo, Norway; 2 Vestre Viken Hospital Trust, Drammen, Norway; 3 Department of Psychology, The Faculty of Social Sciences, University of Oslo, Oslo, Norway; 4 Norwegian Institute of Public Health, Oslo, Norway; 5 Department of Health Promotion and development, The Faculty of Psychology, University of Bergen, Bergen, Norway; 6 Regional Centre for Child and Youth Mental Health and Child Welfare-West, NORCE Norwegian Research Centre, Bergen, Norway

**Keywords:** Opioid-exposed children, Mental health, Foster care, Health care services, SDQ

## Abstract

Given the concerns raised regarding the effects of prenatal exposure to methadone and buprenorphine on the developmental outcomes of the children, this study assessed mental health and use of services in a national sample of school-aged children (*N* = 78) born to women enrolled in opioid maintenance treatment during pregnancy, compared with a group of foster children (*N* = 140). The majority of the opioid-exposed children lived with their birth parent(s) at the time of assessment (*N* = 62), while 16 lived in foster homes. Caregivers completed the Strengths and Difficulties Questionnaire (SDQ) and the Reactive Attachment Disorder scale. Teachers completed the SDQ. Three kinds of services were included in measuring service use: school-based education services, child mental health services, and hospital-based habilitation services. The main finding of the study is that children prenatally exposed to methadone or buprenorphine living with their family of origin had significantly better mental health status than their foster-placed counterparts and that of the comparison group of foster children. In addition, the exposed children living at home had less child welfare involvement, and only half of them were using any of the three services measured. The odds for using services increased significantly in accordance with increasing mental health problems, independent of group affiliation, indicating a need-based access to services. In line with other studies, we found that the odds for using one or more services was 2.3 times greater for boys than for girls. Our results contribute to a more-nuanced understanding of the developmental outcomes of prenatal exposure to methadone and buprenorphine, and factors associated with increased service use in groups of at-risk children.

**Supplementary Information:**

The online version contains supplementary material available at 10.1007/s00787-021-01728-3.

## Introduction

As a result of the increase in use of legal and illegal opioids worldwide, many research reports have focused on developmental outcomes in children prenatally exposed to opioids, primarily in the neonatal period [1]. A substantial number of newborns exposed to opioids in utero exhibit neonatal abstinence syndrome (NAS); a postnatal withdrawal condition of irritability and dysregulation in newborns that by itself predisposes for early interaction problems and increased vulnerability and, eventually to postnatal maltreatment [2]. A longstanding debate in the research literature has addressed the question of whether, and by which mechanisms, prenatal exposure to methadone or buprenorphine may impact child behavior [3]. Research so far has produced mixed results on the possible associations between prenatal exposure to opioids and impaired neurodevelopment in early childhood [4, 5]. Studies on long-term effects are inconclusive with respect to developmental sequelae [6] However, there seems to be a pattern linking prenatal opioid exposure to hyperactivity/inattention problems and increased risk of ADHD [7–9], especially in the context of unsupportive caregiving settings where early dysfunctional maternal–infant relationship potentiate the negative effects of the exposure [10]. It is likely that limited socioeconomic resources, psychiatric comorbidity, and parenting stress that are common in women with addiction problems, have a mutually escalating reciprocal interaction with child behavior over time [11, 12]. Research has shown that even though the direct effect of exposure (i.e., NAS) is a marker for later developmental problems [13], these problems may be explained by a number of factors that accompany opioid exposure with independent effects on child developmental trajectories. Interpretation of results is also made difficult due to methodological challenges such as small sample sizes, high prevalence of concomitant drug use, and maternal psychopathology [14, 15].

Despite these difficulties, research into the relationship between maternal opioid use inside and outside treatment and child health engagement is important and has the potential to encourage substance using mothers to engage with health services to ameliorate child protection risk.

Opioid maintenance treatment (OMT) with methadone or buprenorphine has well-documented benefits for opioid-dependent pregnant women by reducing their mortality, morbidity, and the use of other substances, as well as increasing treatment stability [16]. Treatment stability leads to improved pregnancy follow-up/prenatal care, which can enhance positive maternal health behaviors and parenting skills. Although OMT in pregnancy has clear benefits over continuous drug use and associated lifestyle and mental health problems, maternal use of opioids as methadone/buprenorphine may not be without risks for infant and child development [17]. Studies have shown that unlike pregnant women in the general population, women in OMT do not quit smoking during pregnancy [18]. Smoking is associated with reduced birth weight and length, and may potentiate the negative effects of methadone and buprenorphine exposure in the fetus [19]. In addition, women in OMT may still face pervasive economic, social, and psychological challenges similar to the problems of opioid-dependent women outside treatment.

The relationship between substance abuse and child maltreatment is well recognized by child welfare professionals. Also, health care professionals in the field of substance use treatment are increasingly aware of the special needs of women with parenting responsibilities. The Norwegian approach to OMT is based on an integrated program of follow-up and psychosocial therapy in the context of a state-provided healthcare system, including treatment regulated by national guidelines [20]. Children born to mothers in OMT shall be offered a standard follow-up program that monitors their development and health through infancy and preschool years, without involving the Child Welfare Services (CWS) when the rearing environment is considered sufficiently safe and stable. Pregnant women in OMT who use illicit substances can be court sentenced to mandatory residential treatment to protect the fetus. These women are also subjected to close healthcare surveillance after delivery and the threshold for removing the child from home is low, compared to most other countries’ practices. Norwegian studies show that most women enrolled in OMT during pregnancy were able to abstain from illicit drug use during pregnancy, and 2, 4, and 8 years after delivery [21–23]. However, the same studies show elevated maternal mental and physical health problems and psychosocial difficulties—factors associated with CWS interventions and removals of children from home. Two small-scale Nordic studies indicate that young children of mothers in addiction treatment run a high risk of placement out-of-home [24, 25]. In both studies, the reason for removing children from home was maternal relapse to drug use. In comparison, in our own longitudinal study, 30 of 35 children of mothers in OMT remained at home when assessed at age 4½ [22]. This suggests that compliance in an OMT program may protect children from out-of-home placement, but little is known about the mental health status of these children as they grow into school age and adolescence [26, 27]. So far, research suggests elevated levels of behavior problems in children exposed to opioids as they grow into school age and adolescence [28], but no studies have specifically addressed the mental health outcomes of methadone/buprenorphine-exposed children, compared with other at-risk groups of children from comparably disadvantaged backgrounds. Because children of mothers in OMT are at risk for out-of-home placements, these children constitute a specific subgroup which carries the double burden of both prenatal exposure, separation from biological mothers, and other known risk factors for non-optimal development. From a public health perspective, it is important to explore the clinical- and service use needs of this double-risk group.

Another group of children who are at risk for developmental and behavior problems are foster-placed children who have experienced maltreatment [29]. A Norwegian cross-sectional study of foster children aged 6–12 years documented high rates of mental disorders, partly associated with adverse experiences prior to placement, but also to placement history itself [30]. For half of the children in the study, parental drug use was one of several indicators of maltreatment and subsequent out-of-home placement. Considering the rates of mental health problems among opioid-exposed and foster children [31, 32], there is clearly a need for service provision in these groups. Also, they have specific service needs and use mental health services more often than youth in the general population [33, 34]. In Norway, studies find high service utilization among foster children and their families, where service use is highly (and appropriately) associated with need, in terms of mental health problems both in childhood and adolescence [35, 36].

Accounts of young children entering foster care due to pre- and postnatal parental drug use and how they differ from other foster children without exposure with regard to service use is lacking in the literature. Both groups of children have experienced maltreatment and are at risk for aberrant development. Studies have shown that among youths who were subjects of child welfare investigations or placed in foster care, nearly one half exhibited clinical need [36, 37]. If opioid-exposed children use more services, this may indicate that opioid exposure by itself may act as an independent contributor to the array of risk factors associated with out-of-home placements. A number of foster children suffer from serious problems with social relationships. Social neglect, the absence of adequate caregiving during childhood, and frequent transitions between homes are criteria for Reactive Attachment disorder (RAD) and Disinhibited Social engagement Disorder (DSED) as defined in the diagnostic classification systems ICD-10 and DSM-5 [38]. Also, increased risk for attachment problems characterized by, for instance, indiscriminate friendliness have been identified among children living with drug use in their families [39, 40], similar to the problems found among foster children [41–43]. However, a number of children born to women in OMT remain in parental custody. Studies have shown that home-reared children may also experience inadequate caregiving and may, therefore, be susceptible to disordered behaviors regarding attachment and social engagement [44, 45].

Based on the knowledge that prevalence of mental health problems in drug-exposed and foster care populations is expected to be high, the aim of the present study is to examine and compare the symptom profiles of mental health problems of three groups of school-aged children: (a) opioid-exposed children who live with biological parents, (b) opioid-exposed children placed out-of-home, and (c) children in foster care. The choice of assessment in early school age is motivated by the fact that transition from kindergarten to school implies a major upheaval for most children and in particular for at-risk children when they are required to increasingly self-regulate, focus, and participate independently in a range of activities during the first year of school. As children may behave differently across contexts, informants from different backgrounds sometimes vary in what they perceive to be behaviors that warrant concerns. Children spend a considerable time in school, and certain problems may be present in school but not at home or vice versa. Teachers are commonly used in the present-day research of children’s emotional and behavioral problems [46]. Therefore, we include both caregivers and teachers as informants in this study.

The aims of the current study are:

1. To explore the distribution and level of different mental health problems among opioid-exposed children living with biological parent(s) or in foster care compared to those of non-opioid-exposed foster children.
  (i) As a secondary aim, to assess possible differences between teacher and caregiver reports, regarding mental health profiles for the three groups.
2. To describe service utilization among opioid-exposed children compared to foster children.
  (i) A secondary aim is to investigate the association between mental health problems and service utilization, adjusted for group affiliation.

## Methods

### Participants and procedures

Data for this study were collected from two independent samples. The first sample comprises children born to women enrolled in OMT (78 children) who had previously participated in two separate studies. Sixty two of the children were living with their biological parents (OMT home group) and sixteen children lived in foster care (OMT foster care group). The second sample is a comparison group of 140 foster children where caseworkers did not report any parental drug use prior to placement. These children were presumably not exposed to opioids prenatally.

#### The opioid-exposed exposed children

This dataset contains data on children born to mothers in OMT between 2004 and 2009 who were recruited from two follow-up studies concerning women in OMT and their children, conducted at the Norwegian Centre of Addiction Research. A selection of outcome measures was used in both studies. One study cohort consisted of 36 children born in 2005 and 2006. These participated in a prospective longitudinal study with several assessments through infancy and childhood. Outcomes such as quality of mother–infant interaction, behavioral adaptation, executive functions, and drug use during pregnancy have been previously published [47–49]. This longitudinal cohort included 80% of all children born to mothers in OMT in Norway during those 2 years. Here, we use data from the last assessment, carried out between the years 2013–14, when the children had reached school age (> 6 years). The other cohort consisted of 44 children born in 2004, 2007, 2008, and 2009 of women enrolled in OMT. These women were originally recruited to a study of pre- and post-pregnancy outcomes [50]. A follow-up of this group was done in 2016–17, when the children were of similar age as the longitudinal cohort from 2005–2006. Hence, all children except those born in 2004 were 6–8 years when included in this study, which is typically the age of second and third graders in Norway. Merged data from the two cohorts of opioid-exposed children amounted to information from 76 caregivers and 78 teachers, reporting on 78 children collected between 2013 and 2017.

Information from the mothers was obtained through self-reports and a semi-structured telephone interview conducted by one of the authors (GWS). The interview was presented as a purpose-made questionnaire and consisted of separate sections: a maternal section with questions about well-being and drug use and a child section where mothers reported on services used both for themselves and for the child. Services were reported as “did you ever have contact with”: CWS, education, child mental health, or habilitation services and “do you have ongoing contact with” the same services.

Mental health data were collected from caregivers and the children’s teachers independently, through a secure online questionnaire (the Developmental and Wellbeing Assessment (DAWBA). Because more than half of the children were living in single-parent households with the mother as the primary caregiver, only information given by mothers and three single fathers in the OMT group is reported.

#### The foster care children

The comparison group is a sample of foster children from a larger study [51]. Data collection lasted from September 2011 to February 2012. The inclusion criteria were being aged 6–12 years (the age range for elementary school in Norway), and living in foster families for at least 5 months following legally mandated placement. Foster parents, teachers, and municipal child welfare caseworkers were invited to participate as informants by completing an online survey (for detailed recruitment procedures, see [32]). Children without reported drug exposure (*N* = 140) were selected for the purpose of the present study. This sample are referred to as the “Foster care group”.

#### Combined dataset for this study

For the purpose of the present study, data from the foster care group were pseudonymized before merging it with the dataset from the OMT studies, in accordance with Norwegian legislation (see also Ethics). In this process, three variables were partly aggregated: age (6–7, 8–10, and 10–12); service use (yes/no); and placement information (0, 1, 2, or more placements). Cases from the foster care sample were merged with the OMT data file for samples where the SDQ was completed by both teacher and caregiver. This resulted in data from a total of 218 children. For children 10 years or younger, caregivers also completed the DAWBA Attachment Disorder (RAD) section. Seventy-two percent of the Foster care group were aged 10 years or younger, and were eligible to be assessed with the RAD section, as were 75% of the OMT home group and 75% of the OMT foster care group. Hence, the DAWBA RAD section scores were obtained from caregivers of 132 of children below 10 years.

## Ethics

Ethical approval for the study for the opioid-exposed children was granted by the Regional Committee for Medical and Health Research Ethics (2013/1606/REK Sør-Øst B) and by the Data Inspectorate in Norway. The comparison foster care group is derived from a study approved by the Regional Committee for Medical and Health Research Ethics (2010/2367-1) Western Norway. The Ministry of Children, Equality and Integration provided exemptions from confidentiality for caseworkers, foster parents, and teachers participating in the latter study. For the purpose of combining data from the two studies, anonymization methods were employed, and the data were converted into aggregated statistical format for secondary analyses. The procedure was vetted by the data protection officer of the project-managing organization. Caregivers were not compensated for participation. In the foster care study, teachers were compensated with 28 Euro for participating. In the OMT study, teachers received a gift card worth 20 Euro. Study procedures were in accordance with the Helsinki Declaration. All participating mothers signed written, informed consent at the point of data collection. Participants in the OMT groups had previously consented to being approached regarding participation in upcoming follow-up studies performed by the research team. Participants were explicitly informed about their option to withdraw from the study at any point.

## Measures

The Developmental and Wellbeing Assessment (DAWBA) is a web-based diagnostic interview which covers a spectrum of diagnostic areas, the child’s problems and resources, family background, etc. The DAWBA was completed online by caregivers and teachers at home on their own computers after the receipt of individual access codes. In the current manuscript, we include two sub-sections of the DAWBA: (1) mental health (The Strengths and Difficulties Questionnaire, SDQ), and (2) symptoms of inhibited and socially indiscriminate behavior (DAWBA RAD section).

### Mental health problems

The Strengths and Difficulties Questionnaire (SDQ) is a 25-item questionnaire assessing mental health of 3–16 years old, and may be completed by caregivers and teachers, and by youth from the age of 11 [52]. The SDQ has been reported to be a psychometrically sound measure of overall child mental health in many studies [53, 54], including studies with foster children [55]. The SDQ consist of five subscales. The informant is presented with statements (e.g., “often has temper tantrums” or “has at least one good friend”) and asked to what degree they agree (not true, somewhat true, certainly true). A “Total difficulties score” is also computed and acts as a composite score of the emotional, peer, conduct, and hyperactivity/inattention problem scales, ranging from 0 to 40. Scores above 16 indicate that the child may have a clinically meaningful level of mental health problems in need of further attention [53]. The SDQ also comprises an impact score (0–10) measuring distress to the child and interference of problems to the child’s everyday life. Previous studies have indicated good psychometric properties of the SDQ [53].

### Symptoms of inhibited and socially indiscriminate behavior

The parent version of the Developmental and Wellbeing Assessment (DAWBA) includes a section assessing symptoms of attachment disorders according to DSM-IV criteria, applicable for the age range 5–10 years [56]. This section comprise 14 items rated on a three-point scale according to levels of concern: none = 0, a little = 1, and a lot = 2 [57]. Items are organized in an emotionally withdrawn/inhibited (RAD) subscale of five items with a score range 0–10, and an indiscriminately social/disinhibited (RAD) subscale of nine items with a score range of 0–18. Confirmatory factor analyses have identified a good fit of a two-factor model, congruent with the DSM-5 definition of RAD and DSED, in a sample of foster children [44]. Furthermore, these findings also lend support for the DSM-5 conceptualization of RAD and DSED as separate dimensions of child psychopathology, separate from the four dimensions of more common mental health problems measured by the SDQ. In that study, internal consistency for the inhibited subscale was rather low (Cronbach’s alpha = 0.6), and consequently, we also investigated this aspect of the scales in the current study.

### Reported service use

Information on service use was obtained through the telephone interview based on the structured questionnaire and orally reported by caregivers in the OMT group. Information on service use in the foster care group was provided by caseworkers on a questionnaire. Participants were asked whether they had “ever used” any of the following services (coded yes/no): (1) child and adolescent mental health services (CAMHS); (2) educational psychology services, and (3) hospital-based habilitation services. Information on service use was missing in 24.3% of the cases, but information regarding any service use was only missing for 13% of the respondents. The service use variable was dichotomized in the process of combining the datasets. Note that contact with CWS was not included in the service use variable, as all children in foster care have CWS involvement. However, contact with the CWS for informants in the OMT home group was coded separately as “contact now” (yes/no) and “ever had contact” (yes/no) and is described in the results.

#### Living arrangement and placement history

Of the 62 children residing with biological parent(s), 23 had experienced out-of-home placements. The definition of “any placement history” in the custom-made structured interview was very broad. Changes in living situation for more than a few weeks were categorized as “a placement”. Importantly, the typical placement was short and occurred early in life. Only 5 of the 23 children lived away from their primary caregiver after the age of 3. Twelve children lived away from the primary caregiver ≤ 3 months. Two children moved permanently to their fathers, and seven were placed temporarily with family members. Twelve of the non-family placements lasted for 1–3 months. The remaining two for 5 months at the ages of 1.5 and 3 years. Importantly, during these types of short-term placements, the Norwegian child care system ensures extensive contact between children and care givers. To assess whether these children differed on mental health status, a sensitivity analysis was conducted (see analyses).

Information about placement history was available for all children in the OMT group and 118 children in the Foster care group. For the latter group, the caseworkers provided information about the number of placements children had experienced before the current placement. Information on 22 of the 140 children (15.5%) was missing.

## Analyses

Descriptive demographic and placement variables are presented as group-wise percentages, means, and standard deviations (Table 1, Results). As part of the process of merging the foster care and OMT data, age was collapsed into three groups: 6–7, 8–10, and 11–12 (age group). Information about service use was summed across services per participant and dichotomized (see Ethics) (corresponding to having ever used any of the services). Placement information was aggregated into three levels (see Table 1). Analyses of variance robust to heterogeneity of variances and large differences in sample sizes (Welch test) were used were to compare SDQ and RAD mean total scores and subscales among the three groups (aim 1). Significant *F* tests were followed with pairwise post hoc tests with Games–Howell adjustments for multiple comparisons. Pearson’s *r* was used to test the association between caregiver/teacher pairs. Paired-samples Welch’s *t* tests were used to test the difference in the mean SDQ total scores for caregivers and teachers. Service use for the three groups is presented in Table 1. A logistic regression was used to assess the other variables impact on service use. Each independent variable [Group, AgeGroup, Gender, Placements (grouped), and total SDQ score] was regressed on service use to obtain unadjusted ORs. The RAD data were only present for children < 11 years and were not included. All variables were added into a final model and adjusted odds ratios presented (Table 2). The inferential goodness-of-fit test is the Hosmer–Lemeshow was used to test the null hypothesis that the model is a sufficient fit for the data. Data were analyzed using IBM SPSS Statistics, version 26.0.

### Sensitivity analyses

Due to heterogeneity in age and placement history in our data, two sensitivity analyses were performed. An Independent samples Welch’s *t* test was used to assess potential differences in mental health (SDQ) between the children with placement history (*n* = 23) versus those that had never been placed (*n* = 39), and to assess the probability of group differences. The difference was not significant, and an additional Bayes Factor test was used to quantify probability of a “true” null difference. This showed moderate evidence in favor of the H_0_ (no differences between the groups) BF_01_ = 3.24. In other words, the data are 3.24 times more likely given no group differences (H_0_) than under the alternative hypothesis of different group means). In light of this result, the children with temporary placements out-of-home were analyzed together with those with no placements in the OMT home group. Furthermore, to assess if the inclusion of adolescent children had a significant impact on the results regarding mental health, the SDQ total analyses was performed once without including adolescents (11–12 years old, *n* = 66)—this did not alter any statistical results or interpretations.

## Results

Characteristics of the children in each group are presented in Table 1. There were no significant differences in gender distribution between children living in biological homes and those placed in foster care (see Table 1). Relative to group size, there were more children aged 10 or younger in the Foster child group (*p* < 0.001), which is the upper age for administrating the DAWBA RAD section.

**Table 1:** Sample characteristics and placement history

	OMT home *N* = 62	OMT foster care *N* = 16	Foster care *N* = 140	statistic	*p*
Child female gender, % (*N*)	51.6 (32)	31.3 (5)	45.7 (64)	*Χ*^2^ = 2.18	0.37
Age groups % (*N*)				*Χ*^2^ = 39^b^	< 0.001
6–7 (*n* = 70)	21 (13)	25.0 (4)	37.9 (53)		
8–10 (*n* = 76)	64.5 (40)	50.0 (8)	33.6 (47)		
11–12^a^ (*n* = 72)	14.5 (9)	25.0 (4)	28.6 (40)		
Placements % (*N*)				*X*^2^ = 130	< 0.001
0	62.9 (39)	–	–		
1	32.3 (20)	–	25.7 (36)		
2 +	4.8 (3)	100 (16)	58.6 (82)		
Missing	–	–	15.7 (22)		
Service use^c^ % (*N*)					
No	46.8 (29)	18.8 (3)	24.3 (34)	*X*^2^ = 6.7	0.034
Yes	50.0 (31)	50.1 (8)	59.3 (83)		
Missing	3.2 (2)	31.3 (5)	16.4 (23)		

*OR* odds ratio

^a^note that this group is not included in analysis of the RAD section of DAWBA, which is only administered for children 5–10 years

^b^Chi-square for AgeGroup*Group. Placement information was missing for 22 children in the foster care group

^c^the service use variable indicates having ever used one of three specific services (yes/no), 13% of these data were missing, total *n* = 188: *N* = 60 OMT home, *N* = 11 OMT foster care, *N* = 117 Foster care

Table 1 also gives an overview of the previous placements experienced by the children in the three different groups. Of the OMT home children, 23 (37%) had experienced one or more placements during their lifetime—although they lived with their birth parents at the time of assessment (see also section on living arrangement and placement history above). Reports from either teacher (*n* = 6) or caregiver (*n* = 2) were missing from the OMT data.

### Mental health problems reported by caregivers and teachers

For caregivers, the one-way Welch’s ANOVAs for total SDQ as well as all subscales were significant (SDQ total *p* < 0.001, all subscale *p*’s < 0.011). Table 2 presents comparisons of the mental health profiles of the three groups as reported by caregivers. Overall, the OMT home group had lower rates of mental health problems both compared to the OMT foster care group and the Foster care group as reflected by significant lower scores on emotion, conduct, hyperactivity/inattention, and peer problem subscales. For prosocial functioning in children living at home had significantly higher scores than the non-exposed foster care group, but not the OMT foster care children. Impact score, indicating perceived impairment in everyday functioning, was significantly lower in the OMT home group compared to non-exposed foster care children. There were no significant contrasts between the two foster care samples.

**Table 2:** Between-group comparisons ratings of the Strengths and Difficulties Questionnaire (SDQ) total- and subscales for caregivers and teachers

Group	OMT home (A)	OMT foster care (B)	Foster care (C)	Post hoc Games–Howell corr		
	*N* = 60	*N* = 16	*N* = 140	*p*AB	*p*AC	*p*BC
Caregiver						
** SDQ total difficulties, M (SD)**	8.5 (5.6)	16.3 (6.3)	14.9 (7.8)	**0.001**	**< 0.001**	0.688
Emotional	1.7 (1.7)	3.2 (2.0)	3.6 (2.5)	**0.027**	**< 0.001**	0.738
Conduct	1.4 (1.3)	2.8 (2.0)	2.8 (2.2)	**0.048**	**< 0.001**	0.994
Hyperactivity/inattention	3.7 (2.5)	6.8 (2.6)	6.0 (2.8)	**0.001**	**< 0.001**	0.492
Peer problems	1.7 (2.0)	3.6 (2.4)	2.6 (2.2)	**0.021**	**0.013**	0.296
Prosocial	8.1 (1.8)	6.8 (2.1)	7.0 (2.2)	0.067	**< 0.001**	0.690
** Impact score**	1.3 (2.4)	2.8 (2.7)	2.6 (2.6)	0.145	**0.003**	0.988
	*N* = 56	*N* = 16	*N* = 139			
Teacher						
** SDQ total difficulties, M (SD)**	9.3 (6.7)	14.1 (7.9)	11.9 (7.2)	0.082	0.052	0.507
Conduct	1.3 (1.6)	2.6 (2.5)	1.9 (2.0)	0.091	**0.045**	0.423
Hyperactivity/inattention	4.3 (3.2)	6.3 (3.2)	5.5 (3.1)	0.080	**0.037**	0.614

The letters in parentheses in the group names refer to the letters used in illustrating groups included in the statistical comparisons. “*p*AB” is the *p* value of the Games–Howell corrected test comparing column A (OMT home) and column B (OMT foster care). Bold font denotes statistical difference (α = 0.05)

For teacher reports, omnibus group differences were found for the total difficulties SDQ as well as the conduct and hyperactivity/inattention subscales (all *p*’s < 0.026). Pairwise comparisons showed that these scores were significantly lower in the OMT home group compared to the two groups of children in foster care (see Table 2).

### Informant-based differences of total difficulties scores

**Fig. 1:**
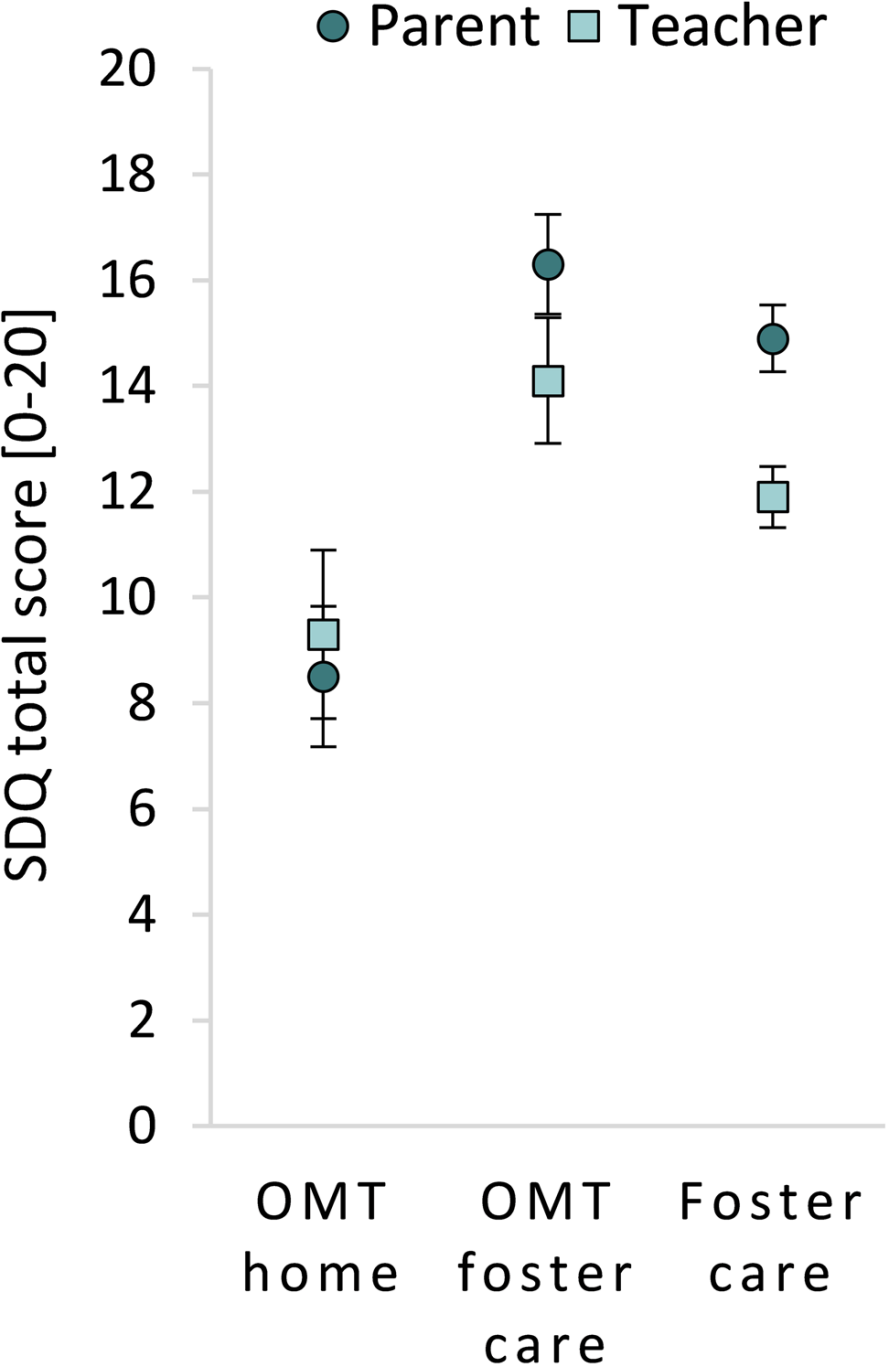
Means and 95% confidence intervals for caregivers (circles) and teachers (squares)

The differences between caregivers and teachers in total difficulties scores are illustrated in Fig. 1 by means and confidence intervals. The results of the paired-samples *t* test are reported in Supplementary Table 1. We found no differences in total difficulties scores between teachers and caregivers for the two OMT groups. Medium-to-large correlations were found between caregiver/teacher pairs in all three groups: OMT home: *r* = 0.43 (*p* = 0.001); OMT foster care: *r* = 0.71 (*p* = 0.002); and Foster care: *r* = 0.58 (*p* < 0.001).

### Symptoms of inhibited (RAD) and socially indiscriminate (DSED) behavior

Table 3 shows the mean scores on inhibited and socially indiscriminate symptom scales for children 10 years and younger. There were significant group differences on all scales (all *p*’s < 0.001), where the OMT home group had lower levels of attachment difficulties symptoms, compared to both foster care groups. In the current study, Cronbach’s alpha for the DAWBA RAD was 0.53 for the five inhibited items (RAD), and 0.82 for the nine socially indiscriminate behavior items (DSED).

**Table 3:** Between-group comparisons of caregiver ratings of RAD scores

Group	OMT home (A)	OMT foster care (B)	Foster care (C)	Post hoc Games–Howell corr		
	*N* = 46	*N* = 11	*N* = 75	*p*AB	*p*AC	*p*BC
RAD/DSED Total	3.5 (2.7)	8.8 (3.0)	9.3 (4.9)	** < 0.001**	** < 0.001**	0.901
RAD inhibit	0.7 (1.0)	3.0 (1.4)	1.9 (1.8)	**0.001**	** < 0.001**	0.070
DSED disinhibit.^a^	2.8 (2.2)	5.8 (3.3)	7.4 (4.1)	**0.031**	** < 0.001**	0.328

The letters in parentheses in the group names refer to the letters used in illustrating groups included in the statistical comparisons. “pAB” is the p value of the Games–Howell corrected test comparing column A (OMT home) and column B (OMT foster care). Bold font denotes statistical significance (α = 0.05)

^a^Data from the inhibited subscale were missing for one participant in the OMT home group (*n* = 45)

### Service utilization

Forty-seven percent of the caregivers in the OMT home group reported no use of any services for their children, compared to 19% of the OMT foster care group. In the Foster care group, the reported percent service use was similar to the OMT Foster group (no service use: 24% and one or more services: 59%) missing data were higher in reports from foster parents (OMT foster care 31.3% and Foster care group 16.4%) compared to biological caregivers (OMT home = 3%). The extent of contact with CWS depended on the children’s living arrangements: while foster-placed children have CWS involvement by definition, 75.8% of the caregivers in the OMT home group reported ever having had contact with CWS, but at the same time, 74.4% disclosed no current involvement with this service.

### Associations between mental health symptoms and service utilization

Table 4 presents unadjusted and adjusted odds ratios for service utilization, depending on group status, gender, age groups, SDQ total difficulties, and number of placements. The full model containing all independent variables was statistically significant *X*^2^ (7, *N* = 125) = 46.0 *p* < 0.001, indicating that the model was able to distinguish between respondents who reported use of services and no use of services. RAD scores were not added to the final model, because these scales were only administered to a subsample of children and, therefore, reduced the sample size. Male gender and higher SDQ total difficulties score increased odds of utilizing at least one service. The odds of utilizing services was more than doubled in boys compared to girls (OR 2.31, CI 1.19–4.48), while the odds for using services increased by 9% for each additional point on the SDQ total difficulties score (OR 1.09, CI 1.03–1.15) when adjusted for the other variables in the model. Age group, group affiliation, and placement were not significantly associated with service utilization in the adjusted model. The Hosmer–Lemeshow test was non-significant, *X*^2^ (7), = 3.7, *p* = 0.882, allowing us to accept the null hypothesis that the model is an adequate fit for the data, and the model correctly identified 72% of the cases.

**Table 4:** Unadjusted and adjusted models predicting service utilization among 2 groups of OMT children, compared to the foster care group

	Unadusted OR (95% CI)	*p*	Adjusted OR (95% CI)	*p*
Foster care **(ref)**				
OMT foster care	1.09 (0.27–4.37)	0.901	0.92 (0.21–4.12)	0.937
OMT home	0.44 (0.23–0.834)	**0.012**	0.78 (0.24–2.54)	0.742
Sex (ref. female)	**2.37 (1.29–4.38)**	**0.006**	**2.31 (1.19–4.48)**	**0.014**
Age 6–7 **(ref)**				
Age 8–10	1.34 (0.68–2.63)	0.399	1.57 (0.73–3.40)	0.201
Age 11–12	2.45 (1.03–5.86)	**0.044**	2.24 (0.87–5.73)	0.072
SDQ total	**1.11 (1.06–1.16)**	** < 0.001**	**1.09 (1.03–1.15)**	** < 0.001**
Placements 0				
Placements 1	2.96 (1.24–7.08)	**0.014**	1.68 (0.54–5.26)	0.372
Placements 2 +	2.29 (1.07–4.94)	**0.034**	1.25 (0.32–4.96)	0.749

OR with *p* values < 0.06 are in bold types *N* = 60 OMT home, *N* = 11 OMT foster care, *N* = 117 Foster care

*OR* odds ratio

## Discussion

A major finding in this study was that children prenatally exposed to methadone or buprenorphine who currently live in their family of origin had significantly better mental health status than their foster-placed counterparts and also than children in foster care without known prenatal exposure to opioids. Considering the large number of scientific reports documenting the high prevalence of developmental problems among opioid-exposed children [58, 59], this result contributes to an important nuancing of previous descriptions of this at-risk group. For instance, a Norwegian hospital-based follow-up study of school-aged children prenatally exposed to alcohol and other substances (including methadone and buprenorphine) reported high rates of mental health problems on all SDQ scales [60], which is markedly higher than in our study for both groups of opioid-exposed children (OMT home and OMT foster care). This discrepancy may be explained by differences in exposure, where the sample in the study of Sandtorv et al. was exposed to a more heterogeneous combination of illicit drugs and alcohol compared with pregnant women in OMT who use very little illicit substances, including alcohol and prescribed benzodiazepines [50]. Another explanation may be the difference in support and follow-up between pregnant women and mothers in the OMT group, compared to that of mothers with different types of addiction problems. The relatively low rates of mental health problems found in our sample of exposed children living with parents may be associated with the mothers’ low use of illegal drugs—but high rates of smoking as we have shown in the previous studies [23, 50, 61]. Considering the negative effects of smoking on the fetus and the developing child, focus on smoking cessation interventions should be a priority in treatment of opioid-dependent pregnant women, similar to what is recommended in the Norwegian national guidelines for pregnant women in opioid maintenance treatment guidelines [20]. Together with commitment to extended prenatal care, healthier pregnancies and better newborn outcomes are promoted, which, in turn, may decrease the risk of subsequent mental health problems in the child.

This being said, it is worth noting that despite better health status of the opioid-exposed children living with their parent(s) compared to out-of-home placed children, they have poorer mental health indicators relative to children in the general population [62]. This finding adds to the previous reviews, emphasizing that children exposed to substances in utero have elevated risks of problems in infancy and childhood [31, 59, 63].

In this study, both caregivers and teachers reported mental health on the same screening instrument (SDQ). A multi-informant approach where data are obtained from parents, teachers, and children themselves is the most prevalent strategy for assessing contextual variations in mental health in children and youth [64]. However, questions can be raised about the validity of parent-reported information. Reports about own child are subjected to idiosyncratic influences, including factors of personal history, education and demographic characteristics, and possible psychological functioning of the responder. For instance, the tendency of caregivers with mental health issues to report higher levels of negative child behaviors or lower levels of positive child behaviors than an independent observer of the same child in the same situation has consistently been showed in the literature [65–67]. Except for the OMT home group, results showed differences between groups similar to other findings where teachers consistently report low symptom loads compared to caregivers, both in normative and risk samples [68, 69].

While caregiver ratings of problems are typically higher than teacher ratings [62], the association between caregiver and teacher-reported symptoms for the OMT children living at home was found to be the opposite direction of foster-placed OMT children, but there was no significant nor relevant difference. Still, questions can be raised around informants’ perspectives and possible rater biases such as socially desirable responding. Caregivers of the opioid-exposed, home-reared children in this study who have had experiences with child welfare might be keen to prove themselves as adequate parents. They may also fear that information about child mental health problems may lead to child welfare involvement with foster care placement as a possible result. Subsequently, social desirability bias could reflect the fear of disclosing negatively loaded information about child functioning and may lead to underreporting on the SDQ. Because some of the children in the OMT home group had experienced out-of-home placements, caregiver information may not be as reliable as for children who has resided with parents all along. However, in cases with short-term, temporary placements, there is close cooperation with child welfare and biological parent to exchange information and monitor child development.

Social desirability bias is not likely an issue for teacher reports in the OMT home group, because many teachers were unaware of the child’s prenatal history with exposure. Hence, teachers’ ratings may be interpreted as a less-biased report compared with the parents’ perceptions of child symptoms, especially in the context of the classroom situation where sustained attention and impulse control are key demands. Finally, it is important to keep in mind that the comparative assessments teachers make about a specific child in a school setting are different from the caregiver’s assessment of the same child in a more transparent home setting. This difference may impact the interpretations of scores on a questionnaire like the SDQ.

Another finding is that the OMT children living with biological parent(s) were rated with lower attachment-related problems (RAD) than both the OMT foster care and the comparison foster care group. This result must be interpreted with caution because of the small sample size of the OMT foster care group. Still, based on research documenting effects of prenatal exposure [6], we may speculate that children with an innate neurobiological vulnerability may be more susceptible to effects of environmental adversities such as poor parenting and transitions between homes. Numerous studies show that factors in the caregiving environment interact with child vulnerability in producing attachment problems [70], but no direct effects of exposure to methadone/buprenorphine have yet been detected. More research, especially longitudinal studies of opioid-exposed children placed in foster care, is highly warranted.

The next important finding in this study concerns service utilization. Nearly half of the parents in the OMT home group did not take advantage of any of the three services accounted for. They also reported low rates of child mental health problems, which might explain why they did not use more services. Of these families, 25% were currently receiving supportive services offered by child welfare. Supportive services could entail measures such as financial aid, home visits, etc., but could also act as part of a risk and safety investigation. Although mothers in OMT do not necessarily have mandated contact with child welfare services, the need for counseling and contact with educational or child mental health services as the child grow older likely remains for many families. Even though mothers entering OMT have better odds for keeping custody of the child and manage parenting compared to women using illicit drugs, they often have a history of losing custody of older children [71, 72]. Such experiences may impede an individual’s engagement with health and care services.

Finally, we examined a number of factors on the likelihood of using services. The strongest predictor of reporting service use was gender, where boys were more than twice as likely to have used services as girls when all other factors in the regression model were controlled for. There is compelling evidence that the prevalence of disruptive behavior disorders are much higher in boys than in girls also when controlling for other factors. This may indicate that service use is more demanded for male children (for review, see [73]).

Another significant predictor of service use was SDQ total scores. For each additional point on the SDQ, we observe a 9% increase in odds for using services, adjusted for age, gender, placement history, and group. On average, the children in foster care scored 6–8 points higher on the SDQ, which indicates a 54–72% increased risk of using services.

Rather surprisingly, the placement history did not affect service use in this study. Other studies of foster children have repeatedly shown that many transitions between homes affect social and emotional development in young children which in turn is associated with help-seeking [74, 75]. We suggest that this finding may reflect the rather high placement stability in both the OMT foster care and the comparison Foster care group, as shown in Table 1. The low number of transitions differs from reports from other studies as the majority of the out-of-home placed children in our study moved only once or twice prior to the present placement, regardless of age and group affiliation [76] 77. It is standard child protection procedure in Norway to move children into temporary foster homes while preparing a final custody decision. Temporary foster homes are especially trained to provide stability, support, and safety to children for a limited period of time, while child welfare investigations are in progress. Hence, temporary foster care, as a semi-professional device, is considered to enhance placement stability.

## Limitations and strengths

Several limitations should be noted. First, the sample sizes of the OMT groups are small. Future studies with a larger sample of opioid-exposed children of mothers in OMT are highly recommended. Also, the limited number of children in the OMT foster care group may not be representative of other groups of children of mothers enrolled in OMT during pregnancy in Norway given the fact that they were extracted from specific subsamples during a specific time-frame. Second, the results presented here may not be generalizable to other countries, given the unique characteristics of Norwegian OMT as a strict treatment model placing high demands on patients with parenting responsibilities. Furthermore, participating in a longitudinal study with several assessment points over the years, parents of opioid-exposed children were given attention which could have beneficial effects on parenting (a form of the Hawthorne effect) and thereby optimize the mental health and well-being of their children compared to children of non-participating mothers in OMT. At the same time, this was also a strength of the study: long-term contact with a research project resulted in participant commitment and contributed to the high retention rate of the study. Another strength is the composition of the OMT group, which is a national cohort of parents and children and therefore broadly representative of the Norwegian population of children prenatally exposed to methadone and buprenorphine during the years 2004–2008. This is also a study group where mothers only used prescribed methadone or buprenorphine during pregnancy, resulting in healthier newborns in contrast to children prenatally exposed to maternal polydrug use.

There are more diverse and targeted services available and services offered both within and outside the municipality and specialist health care systems that could be captured in this study. However, the three services included in service use here are the most frequently used service types for at-risk children and thereby represent a fair picture of service utilization.

Finally, the multi-informant assessment approach of child mental health strengthens our findings. Meta-analytical findings regarding cross-informant correspondence in reports of children’s mental health show low-to-moderate correlations between parent and teacher both with regard to externalizing and internalizing symptoms [78]. In contrast, we note that respondents in our study show surprisingly high cross-informant correspondence, also shown in a previous study of children placed in foster care [79]. We found a rather low internal consistency for the inhibit subscale (Cronbach’s alpha = 0.53). This is in line with the previous findings [80] where item 4 (avoids emotional closeness) and item 10 (unpredictable at reunion) had rather low (but still acceptable). This may contribute to explain the low Cronbach’s alpha also found in the current study, and raise questions on the appropriateness of these two items in measuring inhibited behavior.

It is important to highlight the fact that service uses in risk groups depend on a number of factors, and causal inferences cannot be drawn from the analyses. Several mechanisms could be at work: first, caregivers may ask for services based on perceived needs, but services might also be imposed based on agency standards and community expectations of adequate parenting, especially when children are under CWS supervision. Second, amount of services available can vary across community setting. The sample of opioid-exposed children in this study is a national sample where people live both in cities and at small places in a sparsely populated country. Hence, services differ largely in their availability, type, and tailoring. Furthermore, it is possible that the boys and children with more behavior difficulties had a higher a priori likelihood of being placed in foster care, which has been previously reported [81].

## Conclusion

To our knowledge, this is the first study comparing mental health status of school-aged children of mothers engaged in long-term opioid maintenance treatment (OMT), to foster-placed children of the same age without known prenatal opioid exposure. Identification of children with emerging mental health problems is critical for both clinicians and health care providers to address needs and instigate targeted interventions. This study has underscored the contention that children prenatally exposed to OMT medications are best viewed as special cases of at-risk children, and knowledge gained from other high-risk groups, in this case foster children, can be informative in studies of opiate-exposed children, as well. The heterogeneity of the opioid-exposed children on measures of mental health problems contradicts the idea that these children are “damaged and doomed to fail”, as commented by Barry Lester in 1995 [82]. The results of the current study encourage more-nuanced perspectives on the consequences of prenatal exposure to methadone and buprenorphine within a national, comprehensive health care model. Our results suggest that within this high-risk group, there are subgroups that warrant extra attention and follow-up. The present findings should not blind us from the reality that a number of children prenatally exposed to OMT medications and stressful psychosocial environments are indeed in risk for developing mental health problems which must be addressed. Optimal intervention requires collaborative assessment and treatment models in which professionals share the same knowledge and have the same treatment goals.

## Supplementary Information

Below is the link to the electronic supplementary material.

Supplementary file1 (DOCX 14 KB)
