# Supplementary material for: Mental health and use of health care services in opioid-exposed school-aged children compared to foster children
Source: Eur Child Adolesc Psychiatry. 2021 Feb 16;31(3):495–509. doi: 10.1007/s00787-021-01728-3 (PMC8940845; doi:10.1007/s00787-021-01728-3)
Supplement: Supplementary file 1 — Supplementary file1 (DOCX 14 KB) [file 787_2021_1728_MOESM1_ESM.docx]

**Supplementary table 1. Comparison of SDQ total scores rated by caregivers and teachers**

|  | Caregiver ratings  mean (CI) |  | Teacher ratings  mean (CI) | *p* |
| --- | --- | --- | --- | --- |
| OMT home | 8.5 (7.17-9.83) |  | 9.3 (7.71-10.89) | .397 |
| OMT foster care | 16.3 (15.35-17.25) |  | 14.1 (12.91-15.29) | .150 |
| Foster care | **14.9 (14.27-15.53)** |  | **11.9 (11.32-12.48)** | **<.001** |
